# Utility of two novel multiplexing assays for the detection of gastrointestinal pathogens – a first experience

**DOI:** 10.1186/2193-1801-2-106

**Published:** 2013-03-12

**Authors:** Pascal Kahlau, Monika Malecki, Verena Schildgen, Christine Schulz, Ingo Winterfeld, Sabine Messler, Frauke Mattner, Oliver Schildgen

**Affiliations:** 1 Institut für Pathologie, Kliniken der Stadt Köln gGmbH, Klinikum der Privaten Universität Witten/Herdecke, Ostmerheimer Str. 200, Cologne, Köln D-51109 Germany; 2 Institut für Hygiene, Kliniken der Stadt Köln gGmbH, Klinikum der Privaten Universität Witten/Herdecke, Cologne, Germany

## Abstract

**Background:**

Cause for gastroenteritis range from viral, bacterial to parasitic pathogens. Rapid Multiplexing techniques like ProGastro_SSCS and xTAG_GPP can detect broad panels of pathogens simultaneously.

We performed a field test with a total number of 347 stool samples from adult hospitalized patients that were tested with the Luminex xTAG GPP assay; of the 157 samples positively tested for at least one pathogen by xTAG GPP a total number of 30 samples was retested with the ProGastro SSCS assay. Assays were compared to standard routine diagnostics.

**Findings:**

Multiplexing significantly reduced the time to the initial identification of a pathogen. Moreover, multiplexing detected pathogens for which a diagnostic assays was not requested by the physician and thus may be an important tool for avoiding nosocomial outbreaks.

**Conclusion:**

This first frontline approach with these assays approves their utility compared to conventional microbiological methods.

## Short-form paper

Nosocomial gastrointestinal infections remain a major health care problem worldwide, can be caused by bacterial, viral, or parasitic pathogens, and require rapid diagnostic identification in order to avoid further spreading, both for medical and economic reasons (Greig & Lee 2012; Vonberg et al. 2008; MacCannell et al. 2011; Zingg et al. 2005). In daily practice, the interaction of different medical disciplines (Virology, Microbiology, and Parasitology), and the combination of conventional microbiological methods with molecular assays can cause delays in identification of gastrointestinal pathogens, thereby hindering efficient counteractions (Mauch et al. 2009). For rapid identification of the pathogens that may cause nosocomial outbreaks a rapid multiplex front line screening assay is highly desirable and would be helpful in infection control (Khamrin et al. 2011). In the case of *C. difficile* infections a rapid identification may not only reduces nosocomial transmission but favor the clinical outcome by initiating more timely a specific therapy (Cohen et al. 2010).

Recently, two novel assays were launched to the market which have the potential to speed up the initial pathogen identification, namely the xTAG Gastrointestinal Pathogen Panel (xTAG GPP) developed by Luminex (Luminex Molecular Diagnostics, Toronto, Canada), and the ProGastro SSCS assay by Gen-Probe (Gen-Probe Incorporated, San Diego, USA). The xTAG GPP is able to identify 15 pathogens; these are: *Adenovirus 40/41, Campylobacter, Clostridium difficile, Cryptosporidium, Entamoeba histolytica*, Enterotoxigenic *Escherichia coli* (ETEC), *E. coli* O157, *Shiga-like Toxin producing E. coli* STEC), *Shigella, Salmonella, Giardia, Norovirus GI/GII, Rotavirus A, Vibrio cholerae and Yersinia enterocolitica*(Malecki et al. 2012)*,* whilst the ProGastro SSCS assay by Gen-Probe (Gen-Probe Incorporated, San Diego, USA) is able to differentiate between *Salmonella, Shigella, Campylobacter* (*C. jejuni* and *C. coli* only) and *Shiga Toxin-producing Escherichia coli* (STEC).

We compared both assays in our clinical routine in order to get a first impression of their utility compared to a “classical” diagnostic algorithm as asked for by the physician (Bacteria: culture methods, *Rota-, Adeno- Norovirus:* ELISA, Parasites: microscopy, *C. difficile* Glutamat-Dehydrogenase-ELISA followed by toxin ELISA and culture in cases ELISA was tested negative). Stool samples were collected routinely and simultaneously sent to the Microbiology/Virology laboratory and to our laboratory where the multiplexing and real-time platforms were located. A total number of 347 stool samples from adult hospitalized patients was tested with the Luminex xTAG GPP assay; of the 157 samples positively tested for at least one pathogen by xTAG GPP a total number of 30 samples was retested with the ProGastro SSCS assay; all samples were routinely screened for gastrointestinal pathogens according the physicians’ requests, which does not necessarily mean that the full pathogen panel available in the xTAG GPP assay was tested by classical methods.

Stool samples were pretreated as requested by the manufacturer’s protocols and subject to nucleic acid extraction by using the Qiagen stool kit (Qiagen, Hilden, Germany) automated on the QiaCube platform. The eluates were subjected to PCR reactions as recommended both by the ProGastro_SSCS and xTAG_GPP assay manufacturers and were processed further on the Rotorgene platform (ProGastro SSCS) or the Luminex machine (xTAG GPP).

In detail, pretreatment for the xTAG GPP and ProGastro SSCS assay using the NucliSENS easyMAG lysis Buffer was performed as follows: 1 mL of NucliSENS easyMAG Lysis Buffer (bioMérieux, Nürtingen, Germany), 10 μL of *E. coli* phage MS2 (Luminex Molecular Diagnostics, Toronto, Canada) for the xTAG GPP or 10 μL of Gastro RNA/DNA Internal Control (GIC) (Gen-Probe Prodesse, Waukesha, USA) for the ProGastro SSCS assay and approximately 100 to 150 μg of stool were added to Precelllys Glas/Keramik-KIT SK38 (PEQLAB Biotechnologie GmbH, Erlangen, Germany) bead tubes. The tubes were vortexed by using the MagNA Lyser instrument (Roche Diagnostics Deutschland GmbH, Mannheim, Germany) at 5000 rpm for homogenization of the tissue. After 10 minutes incubation time the SK38 (PEQLAB Biotechnologie GmbH, Erlangen, Germany) tubes were centrifuged at 14,000 rpm for two minutes at room temperature (RT) using the Microcentrifuge 5424 (Eppendorf AG, Hamburg, Germany). 200 μL of the supernatant were used for the extraction.

For ProGastro SSCS assays, ParaPak Culture & Sensitivity (PP) medium were used for pretreatment as follows: 500 μL of PP medium (Meridian Bioscience, Cincinnati, USA) were introduced into a 1.5 mL tube and 10 μL of the prepared GIC (Gen-Probe Prodesse, Waukesha, USA) were added. The GIC was prepared according to the manufactures instructions. Approximately 100 to 150 μg of stool samples were inserted and the mixture was vortexed for 1 minute. After a short centrifugation at RT 200 μL of the supernatant were used for the extraction. Extraction of DNA from stool samples was performed by using the QIAamp MinElute Virus Spin Kit (Qiagen, Hilden, Germany) for the QIAcube system according to the manufacturer’s instructions. The eluate was stored at −80°C until usage.

Pathogen detection was performed with the xTAG Gastrointestinal Pathogen Panel and the ProGastro SSCS assay as follows: xTAG OneStep RT-PCR Buffer 5X, xTAG RNase-free water, xTAG BSA were thawed at RT and afterwards kept on ice for the Mastermix (MM). 2.5 μL xTAG RNase-free water, 7.5 μL xTAG OneStep Buffer 5X and 0.5 μL xTAG BSA (10 mg/mL) were added to the MM for each PCR reaction. The xTAG GPP Primer Mix as well as the xTAG OneStep Enzyme Mix were thawed and kept on ice during the preparation of the MM. 2.5 μL of Primer Mix as well as 2.0 μL of the Enzyme Mix were added to the MM for each PCR reaction. All reagents were vortexed and briefly centrifuged before use. The volume for one extra reaction was included for ≤ 10 samples, and the volume for two extra reactions were included for more than 10 samples to compensate pipetting variability.

10 μL of the extraction product were mixed with 15 μL of MM in a 0.2 mL thin-walled PCR-tube for a total RT-PCR reaction volume of 25 μL. The negative control contained 10 μL xTAG RNase-free water instead of the extraction product. No extra positive controls were included besides the internal MS2 control.

The PCR tubes were placed in a thermal cycler preheated to 53°C. Four stages were used for the RT-PCR program: The first was 1 cycle at 53°C for 20 min followed by 1 cycle at 95°C for 15 min as the second stage to ensure proper denaturation. Amplification was achieved in the third stage starting at denaturation temperature of 95°C for 30 sec, followed by annealing at 58°C for 30 sec and elongation at 72°C for 30 sec repeated 38 times. This temperature was held in the last stage for 2 min as a final elongation. When the program finished the temperature was set and hold at 4°C at which all samples were stored after RT-PCR.

The xTAG GPP Bead Mix and the xTAG Reporter Buffer were taken out of the refrigerator and brought to RT in the dark. 20 μL of bead mix were aliquot into a 96-well microtitre plate and 5 μL of vortexed RT-PCR product were added. After vortexing and a short centrifugation of the xTAG 0.22 SAPE, 1 μL was taken and diluted in a 2.0 mL tube with 74 μL of xTAG Reporter Buffer for each sample. An additional reaction volume of xTAG 0.22 SAPE and xTAG Reporter Buffer was added for ≤ 10 samples or two additional reaction volumes for more than 10 samples. 75 μL reporter solution were then pipetted in each microtritre well containing 25 μL bead mix and RT-PCR product for a total volume of 100 μL per hybridization reaction. The plate was covered with microseal film and placed in the thermal cycler preheated to 60°C. This was the temperature the program was started at for 3 min, followed by 45 min at 45°C. The temperature was hold at 45°C at the end of the program because the samples may not cool down until analysis which was proceeded directly after the hybridization. The Luminex 100/200 (Luminex Molecular Diagnostics, Toronto, Canada) was used with the TDAS GPP software 1.00. The instrument was started at least 20 min before the hybridization was finished to heat up the laser and the Luminex XYP heater block was set to 45°C prior at least 10 min to analysis to prevent prolonged hybridization times. After hybridization the microseal film was removed from the microtitre plate and the plate was inserted into the heater block. The xTAG GPP protocol was used for each batch and the data produced were analyzed by the xTAG Data Analysis Software (TDAS).

For the ProGastro SSCS assay 0.2 mL thin-walled PCR tubes (Qiagen, Hilden, Germany) were used for setting up the PCR reactions. The volume for the PCR reaction was 25 μL containing 5 μL nucleic acid of the samples or positive controls (*Salmonella, Shigella, C. jejuni,* Shiga Toxin producing *E. coli* (SSCSpc) and *C. coli*) and 20 μL of SSC or STEC mix, respectively. A positive matrix control was not included. The negative control prepared for the quantification step consisted of 5 μL molecular grade water and 20 μL SSC or STEC mix, respectively. All tubes were kept on ice during the preparation for the PCR. A short description of the SSC and STEC mixes can be found in Table 1.

**Table 1:** **Comparison of results acquired by using the xTAG GPP or conventional techniques**

Overall amount of xTAG GPP results compared to results of conventional methods				
104				
Confirmed results	Test was not requested by physician	positive samples produced by xTAG GPP	Discrepancies because of positive results produced by conventional methods	different positive results for both test methods
70	19	6	7	2

104 data sets were either confirmed by both tests, were not performed with a default test as it was not marked at the requisition form or were inconsistent. When the tests were not matching further differentiation was achieved by showing if the positive result was produced by the xTAG GPP, a conventional method or if both laboratories produced positive but not matching results.

The Rotor-Gene Q system (Qiagen, Hilden, Germany) was used to carry out the Real Time PCR analysis. A 36-Well rotor was used to take up a maximum of 36 thin-walled 0.2 mL PCR tubes. The amplification process consisted out of three different cycling stages. In the first the temperature was 95°C for 600 secs with shut down optics to ensure a proper and complete denaturation of the nucleic acid. This stage was not repeated. The second stage started with a denaturation temperature of 95°C for 30 secs and proceeded to an annealing/elongation temperature of 55°C for 60 secs. It was repeated five times and the optics were shut off. The last stages consisted of 40 cycles starting with a denaturation temperature of 95°C for 10 secs with shut off optics and an annealing/elongation temperature of 55°C for 60 secs with running optics.

Four different optical channels were used for detecting the presence of target genes according to the manufacturer: FAM, TET, TxR and Cy5. These channels were named green, yellow, orange and red by the Rotor-Gene Q software (Rotor-Gene 2.0.2.4), respectively.

The thresholds for the ct values were set and analyzed manually for each channel that was measured. The results were compared to the outcomes of the previously test with the xTAG GPP performed with the Luminex 100/200 system.

With the xTAG GPP assay 347 samples were tested and 157 were positive for one or more pathogenic organisms. *Norovirus* GI/GII caused 82 infections which accounts for 52% of all positive samples. The second most abundant organism detected was toxigenic *C. difficile*. In 47 cases both toxins (A and B) were detected, in 11 cases only toxin A, in one case only toxin B. Infections with *Campylobacter* were observed 8 times, infections with *Salmonella* and *Rotavirus* A were detected 4 times for each of the organisms. In 3 cases *Cryptosporidium* was detected, *Shigella*, *Enterotoxigenic E. coli*, *Shiga-like toxin-producing E. coli* and *Giardia lamblia* were all detected once. In one case a double infection with *Rotavirus* A and toxigenic *C. difficile* occurred and was confirmed by tests with the conventional methods (ELISA). Other double infections that were observed included 4 times *Norovirus GI/GII* with toxigenic *C. difficile*, one time *Norovirus GI/GII* with *Salmonella* and one time *Salmonella* with *Giardia lamblia,* but these cases could not be confirmed by conventional methods (culture methods for Salmonella, ELISA and PCR for Norovirus and microscopy for Giardia). For one double infection with *Campylobacter* and *Norovirus GI/GII* only the presence of *Campylobacte*r was confirmed by culture, but not *Norovirus GI/GII.*

Of the total sample cohort it was possible to compare the xTAG GPP to the conventional diagnostic techniques used by our external microbiology lab to screen for diarrheal pathogens in 104 cases for which full documentation was available. In 19 samples the xTAG GPP detected a pathogen that was not being tested for by the conventional method as the physician who requested the diagnostics did not mark the respective test in the requisition form (Table 1). In 70 cases the results of the xTAG GPP were confirmed by the respective conventional method. However there were 15 discrepancies which were further differentiated into 6 positively tested samples by the xTAG GPP that were negatively tested by conventional methods as well as 7 positively tested samples by conventional methods that did not produce positive signals by the xTAG GPP. Furthermore in two cases the pathogens that were displayed were completely different, namely in one case ELISA detected Norovirus whilst xTAG GPP detected C. difficile, and in the other case C. difficile was detected by conventional methods whilst xTAG GPP detected rotavirus. All *C. difficile* positive samples that were detected by conventional methods were also confirmed by either a positive *C. difficile* culture test or by a *C. difficile* toxin ELISA.

Discordant results were in detail (Table 2): A *Norovirus* infection was observed for the first two samples (A and B) followed by double infections with *Norovirus* and *C. difficile* in samples C and D by the conventional techniques (Bauer et al. 2009), i.e. ELISA for Norovirus and culturing for *C. difficile;* none of these results (A-D) was confirmed by xTAG GPP. Single pathogens were detected by conventional culturing in samples E and F, *C. difficile* and *Yersinia*, respectively (Table 2). The following samples G to K did not produce positive signals during conventional tests. The xTAG GPP detected *Norovirus* GI/GII in samples G and H, *C. difficile* in sample I, *Shigella* in J and Stx2 in sample K. Moreover, 29 samples previously tested positive by the xTAG GPP were retested by the ProGastro SSCS method. Except two differences ProGastro SSCS confirmed the xTAG GPP results: In one case, xTAG GPP detected Shigalike-toxin 1 gene which was not found by the ProGastroSSC, in the other case xTAG GPP detected *Shigella* which was detected neither by culturing nor the ProGastroSSC assay. The results are summarized in Table 3.

**Table 2:** **Detected pathogens that were either detected only by xTAG GPP or by the conventional methods**

Sample	Results of the xTAG GPP	Results of conventional methods
A	negative	*Norovirus* GI/GII
B	negative	*Norovirus* GI/GII
C	negative	Norovirus GI/GII + *C. difficile*
D	negative	Norovirus GI/GII + *C. difficile*
E	negative	*C. difficile*
F	negative	*Yersinia*
G	*Norovirus* GI/GII	negative
H	*Norovirus* GI/GII	negative
I	*C. difficile*	negative
J	*Shigella*	negative
K	Stx2	negative
L	Norovirus GI/GII + *Campylobacter*	*Campylobacter*
M	*Campylobacter*	Norovirus GI/GII + *Campylobacter*
N	*C. difficile*	Norovirus GI/GII + *C. difficile*

In case of sample L and in samples M and N double infections were found by the xTAG GPP and the conventional methods, respectively, instead of a single infection which was observed for the opposing test**.**

**Table 3:** **Comparison of ProGastro SCCS with xTAG GPP Assays**

Sample #	ProGastro SSCS	xTAG GPP
1	Stx1/Stx2	**Stx1/Stx2**
2	Stx1	**Stx1**
3	Campylobacter	**Campylobacter**
4	Shigella	**Shigella**
5	Salmonella	**Salmonella**
6	negative	**Stx1**
7	Campylobacter	**Campylobacter**
8	Campylobacter	**Campylobacter**
9	Shigella	**Shigella**
10	Shigella	**Shigella**
11	Salmonella	**Salmonella**
12	Salmonella	**Salmonella**
13	Stx2	**E. coli O157, Stx2**
14	Stx1/Stx2	**Stx1/Stx2**
15	Campylobacter	**Campylobacter**
16	Shigella, Stx2	**Stx2**
17	Stx2	**Stx2**
18	Campylobacter	**Campylobacter**
19	Campylobacter	**Campylobacter**
20	Campylobacter	**Campylobacter**
21	Campylobacter	**Campylobacter**
22	Campylobacter	**Campylobacter**
23	Campylobacter	**Campylobacter**
24	negative	**negative**
25	negative	**negative**
26	negative	**negative**
27	negative	**negative**
28	negative	**negative**
**29**	**negative**	**Shigella**

**Figure 1:**
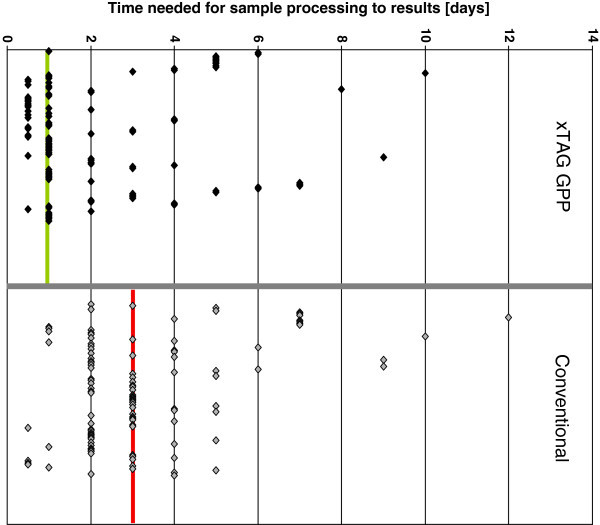
**Time needed from receiving a sample to communicate the diagnostic findings.** The 104 samples handled with the xTAG GPP were compared to the corresponding samples that were tested with conventional methods. For reasons of comparability the median (xTAG GPP: green, conventional: red) was drawn in the diagram.

Most strikingly, the turnover time from sampling to the first diagnostic result was significantly decreased by using of multiplexing assays. The median turnover time using the xTAG GPP assay was 1 day, ranging from 0.5 days to a maximum of 2 days except one sample that was lost for several days due to a mail transportation error, whilst the median turnover time for the classical diagnostic algorithm was 3 days (Figure 1), which was highly significant with a p-value of p = 0.0000021 resulting from the one-sided T-test for paired samples and a p-value of p = 0.0000042 for the two-sided T-test.

The major bias of our study is that only a limited number of samples positive for the respective pathogens was tested and that for some pathogens like *Giardia* not a single positive sample was available. However, despite these limitations the study shows that the turnover time from sampling to the first result may be significantly reduced provided future studies with larger patient cohorts could confirm our present results.

In summary, provided that optimal transport and lab logistics are established, both assays can lead to a result from sampling to pathogen identification within 6–10 hours. This in turn is most important in order to initiate more specific therapy, setup rapid and efficient hygiene counteractions or, vice versa, to de-isolate patients and thus save economic resources due to false or unnecessary blockades of hospital beds. For the decision to de-isolate patients dependent on a negative result of a multiplex-PCR test, negative predictive values are needed in future. For some bacterial pathogens with the ability of differing antibiotic susceptibilities culture methods will be needed in addition. Finally, although it might be to early for this conclusion, it is worth to restructure guidelines for the diagnostic and treatment procedures of gastrointestinal infections that take into account the advantages of early multiplexing technology. It can be concluded multiplexing in gastrointestinal infection diagnostics has the potential to (a) reduce the time to the first identification of a pathogen, may (b) influence subsequent clinical courses by earlier start of specific treatments and diagnostical procedures and avoid false isolations (which in turn is a significant economic factor), and may (c) reduce the number of false negative diagnostics due to missing assays that were not requested after the first round of sampling.
